# Transcriptome and Co-Expression Network Analyses of Resistant and Susceptible Rice Cultivars in Response to *Meloidogyne graminicola*

**DOI:** 10.3390/ijms26115315

**Published:** 2025-05-31

**Authors:** Shirui Zhang, Zitong Xiao, Kexiang Shen, Wentao Lai, Shunbiao Du, Linyan Zhou, Jiansong Chen

**Affiliations:** 1 College of Agriculture and Biology, Zhongkai University of Agriculture and Engineering, Guangzhou 510225, China; shirui_zhang@163.com; 2 Rice Research Institute, Guangdong Academy of Agricultural Sciences, Guangdong Key Laboratory of Rice Science and Technology, Guangdong Rice Engineering Laboratory, Key Laboratory of Genetics and Breeding of High Quality Rice in Southern China (Co-Construction by Ministry and Province), Ministry of Agriculture and Rural Affairs, Guangzhou 510640, China; 15986050091@163.com (Z.X.); skx1604969581@163.com (K.S.); 19045314019@163.com (W.L.); 18856746112@163.com (S.D.); 3 The Laboratory of Seed Science and Technology, Guangdong Key Laboratory of Plant Molecular Breeding, South China Agricultural University, Guangzhou 510642, China

**Keywords:** *Meloidogyne graminicola*, transcriptome, WGCNA, hub gene

## Abstract

*Meloidogyne graminicola* represents a significant pathogen of rice, with considerable variability in nematode resistance observed across rice germplasms. However, the gene regulatory networks and molecular mechanisms underlying the differential responses of resistant and susceptible cultivars to *M. graminicola* infection remain poorly understood. To identify potential sources of resistance, 122 indica cultivars were screened under controlled conditions based on gall formation in infected roots. Notably, Indian indica accession 685 exhibited exceptional resistance, characterized by complete suppression of nematode development within root tissue. To investigate the molecular responses of rice cultivars to *M. graminicola* infection, RNA sequencing was conducted to analyze gene expression profiles at 5 days post-inoculation (dpi) in resistant cultivar 685, moderately susceptible cultivar 1008, and susceptible cultivar 9311. Subsequent differential gene expression analysis and weighted gene co-expression network analysis (WGCNA) identified key biological pathways, including sugar metabolism, autophagic degradation, and phytohormone signal transduction. Additionally, candidate hub genes were identified and validated through RT-qPCR. This study offers new insights into rice–*M. graminicola* interactions, highlighting critical molecular factors involved in resistance and susceptibility in host plants.

## 1. Introduction

Rice (*Oryza sativa* L.), a primary dietary staple for nearly half of the global population, is cultivated across more than 100 countries, with Asia accounting for approximately 90% of the global output [1]. Rice growth is susceptible to various pathogens, including fungi, bacteria, viruses, and plant-parasitic nematodes, resulting in significant annual yield losses [2]. Plant-parasitic nematodes alone account for a 12.3% reduction in global food production annually, equating to an estimated economic loss of USD 157 billion [3]. Approximately 20% of these losses are specifically attributed to rice production [4]. Although over 200 nematode species have been identified in association with rice, only a few species cause substantial damage to rice crops [5]. Among these, the rice root-knot nematode (*Meloidogyne graminicola*) is one of the most prevalent and damaging pests in rice systems, representing a major threat to global rice production [6]. *M. graminicola* infections disrupt root system development, leading to symptoms such as stunted growth, chlorosis, early flowering, premature maturity, and reduced grain yield [7]. Depending on the severity, yield losses due to *M. graminicola* can range from 28% to 87% [8].

During early stages of infestation, the infective second-stage juvenile (J2) of the root-knot nematode utilizes a stylet to traverse plant cells without damaging adjacent ones [9]. Following entry, the nematode migrates toward the root apex and subsequently ascends the vascular bundle to locate a suitable feeding site. Beyond the physical penetration of the stylet, nematodes secrete various effectors that facilitate their movement and the establishment of a feeding structure known as giant cells (GCs) [10]. GCs serve as the exclusive nutrient source for all nematode life stages, and their development correlates with the degree of nematode infection as well as the plant’s susceptibility or resistance [11]. Disease resistance in plants is often mediated by nucleotide-binding leucine-rich repeat (NLR)-type resistance genes (R genes), which recognize pathogen invasion and activate defensive responses [12]. In plant–nematode interactions, immune responses triggered by dominant R genes include the accumulation of reactive oxygen species, callose deposition, and localized cell death, all of which inhibit feeding site formation. Several R genes encoding NLR proteins associated with root-knot nematode recognition have been identified, such as *MG1* in rice, *Mi-1.2* and *Mi-9* in tomato, *CaMi* in pepper, and *Ma* in plum [13,14,15,16]. Conversely, loss-of-function mutations in susceptibility genes (S genes) can confer recessive resistance, limiting the pathogen’s virulence and often providing broad-spectrum resistance [17]. For example, the knockout of the *Mlo* gene in barley confers durable resistance to powdery mildew [18]. Regarding plant-parasitic nematodes, the Arabidopsis gene *AtHIPP27* has been identified as a susceptibility gene for the beet cyst nematode *Heterodera schachtii* [19]. The resistance response mediated by *AtHIPP27* is characterized by the degradation of or failure to form feeding site cells during later stages of nematode infestation without triggering a hypersensitive response [19].

Disease resistance in plants is governed by a range of genetic and molecular mechanisms, including cell wall-modifying proteins, growth hormone-related proteins, reactive oxygen species scavenging systems, and transcription factors [20]. Comparative transcriptomic analyses between durably resistant and susceptible genotypes under pathogen challenges yield critical insights into the underlying molecular foundations of resistance and susceptibility [21]. For instance, in the tobacco–*Meloidogyne incognita* system, transcriptomic profiling uncovered the marked differential expression of genes related to cell wall modification, hormone signaling, reactive oxygen scavenging systems, and transcriptional regulation in susceptible plants [21]. Likewise, studies on resistant and susceptible tomato cultivars infected by *M. incognita* and *Meloidogyne hapla* showed that 25% of genes annotated as phytohormones were modulated by root-knot nematodes (RKNs) [22]. To dissect such transcriptomic complexity, gene co-expression network analysis (GCNA) is a systems biology approach used to identify correlations in gene expression across large datasets. Among GCNA methodologies, weighted gene co-expression network analysis (WGCNA) has emerged as a standard tool for constructing and visualizing co-expression networks modules derived from transcriptomic profiles [23]. WGCNA has become the primary method for identifying and analyzing gene modules related to specific biological processes in various plant species [24]. This approach can be applied to unravel the complex network of plant–nematode interactions. By constructing gene co-expression networks, WGCNA facilitates the identification of gene modules and key genes involved in resistance or susceptibility to plant-parasitic nematodes, offering insight into the molecular responses of plants to nematode infestation. In one example, WGCNA revealed key gene modules from 36 soybean root samples exposed to soybean cyst nematode (SCN) HG Type 0 (race 3), elucidating the transcriptional landscape underpinning plant defense [25].

In this study, phenotypic screening was conducted to assess resistance to rice root-knot nematodes among 122 indica rice varieties using artificial inoculation. Notably, rice accession 685 exhibited high resistance to *M. graminicola*. To explore the molecular basis of nematode resistance and susceptibility, RNA-seq transcriptome analysis was performed on three indica rice varieties: 685 (highly resistant), 1008 (moderately susceptible), and 9311 (highly susceptible). This analysis identified key pathways associated with nematode resistance- and susceptibility-related pathways, notably those involved in sugar metabolism, autophagic degradation, and salicylic acid (SA)/brassinosteroid (BR) phytohormone signal transduction. Additionally, hub genes related to resistance and susceptibility in cultivars 685 and 9311 were identified. These regulatory networks and hub genes are likely critical in modulating nematode parasitism in rice.

## 2. Results

### 2.1. Identification of Resistance to M. graminicola in Indica Rice Cultivars

**Figure 1:**
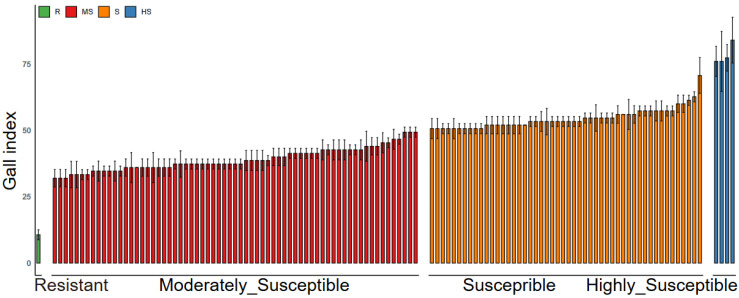
Screening of 122 indica rice cultivars for root-knot nematode resistance. A total of 122 indica rice germplasm resources were evaluated for resistance to *Meloidogyne graminicola* by inoculating each plant with 200 pre-parasitic second-stage juveniles (pre-J2s). Gall formation was assessed at 20 days post-inoculation (dpi), and resistance/susceptibility was classified using the gall index (GI) scoring system: immune (GI = 0); highly resistant (0.1 ≤ GI ≤ 5.0); resistant (5.1 ≤ GI ≤ 25.0); moderately susceptible (25.1 ≤ GI ≤ 50.0); susceptible (50.1 ≤ GI ≤ 75.0); and highly susceptible (GI > 75.0). Bars represent the mean GI ± SD from three independent biological replicates, each comprising five plants. R = resistant; MS = moderately susceptible; S = susceptible; HS = highly susceptible.

**Figure 2:**
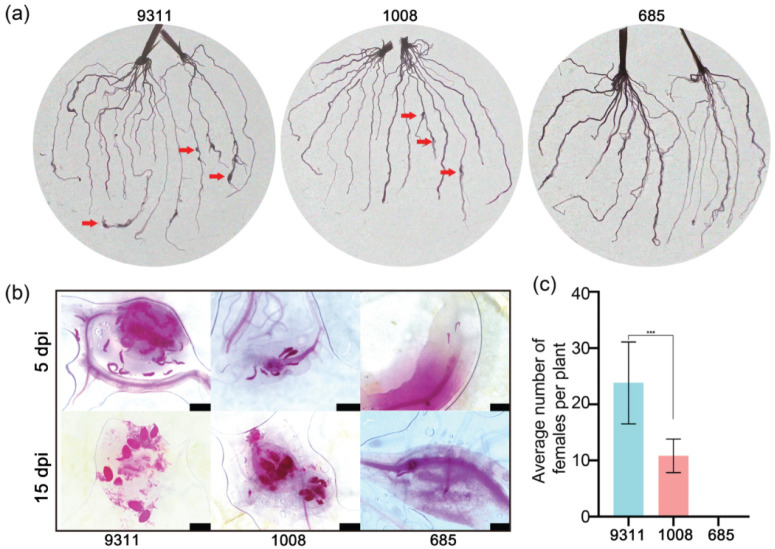
Responses of rice cultivars to *Meloidogyne graminicola* infestation. (**a**) Representative images of root systems from resistant cultivar 685, moderately susceptible cultivar 1008, and highly susceptible cultivar 9311, photographed at 15 dpi following *M. graminicola* infestation. Red arrows indicate root galls. Experiments were repeated three times. (**b**) Nematode development within infected roots was assessed at 5 and 15 dpi using acidic magenta staining to visualize different developmental stages of *M. graminicola* in cultivars 685, 1008, and 9311. Experiments were conducted in triplicate, each with six biological replicates. Scale bar = 50 μm. (**c**) The number of female nematodes was quantified at 15 dpi. Data are presented as mean ± SD from 15 plants across three independent biological replicates. *** *p* < 0.001, Student’s *t*-test. 5 dpi = 5 days post-inoculation; 15 dpi = 15 days post-inoculation.

To identify rice resources resistant to *M. graminicola*, a systematic screening of 122 indica rice varieties was conducted through artificial inoculation under controlled conditions. Root galling and the corresponding gall index were used to assess the host’s suitability for nematode infection. Based on gall index values, cultivars were categorized as resistant, moderately susceptible, susceptible, or highly susceptible (Figure 1). Among the tested varieties, 4 cultivars (3.28%) were classified as highly susceptible, 48 cultivars (39.34%) as susceptible, and 69 cultivars (56.56%) as moderately susceptible. Only cultivar 685 exhibited a high level of resistance to nematodes (Table S1). Based on the gall index, cultivars 685 and 9311 were identified as resistant and highly susceptible, respectively, with cultivar 1008 classified as moderately susceptible (Table S1). Additionally, the tissue staining of rice roots infected with nematodes at 5 and 15 days post-inoculation revealed the presence of galls and mature females in cultivars 9311 and 1008, while no galls or mature females were observed in cultivar 685 (Figure 2a,b). Statistical analysis showed that the average number of female nematodes in cultivar 9311 was approximately double that in cultivar 1008, with an average of 23.80 versus 11.87 females per plant (Figure 2c). To further elucidate the molecular mechanisms underlying nematode infestation in rice with varying resistance levels, transcriptome analysis was performed on the highly resistant cultivar 685, the moderately susceptible cultivar 1008, and the highly susceptible cultivar 9311.

### 2.2. RNA-Seq Data Alignment to the Reference Genome

Differential gene expression analysis was performed on rice cultivars 9311, 685, and 1008 at 5 days post-inoculation (dpi) with and without *M. graminicola* infection, utilizing RNA-Seq datasets. The raw sequencing data were filtered to generate high-quality clean reads, and sequence contaminants were checked using FastQC. A total of 684.27 million clean reads were generated from 30 cDNA libraries, each with five independent biological replicates (Table S1). The GC content of individual libraries ranged from 53% to 56% (Table S1). All clean data were deposited in the NCBI Sequence Read Archive (SRA, accession number PRJNA1240874). Clean paired-end reads (~150 bp) were aligned to the IRGSP-1.0 rice reference genome via HISAT2, achieving mapping rates between 90.09% and 93.65% per sample. This yielded 38,978 identified transcripts (Table S1). Subsequent analysis was conducted using only uniquely mapped reads.

### 2.3. Comparison of the DEGs Between Resistant and Susceptible Rice Cultivars in Response to M. graminicola Infection

**Figure 3:**
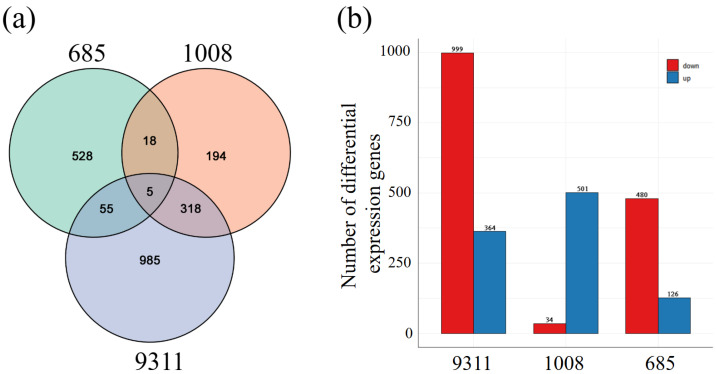
Differentially expressed genes (DEGs) in indica rice cultivars at 5 days post-inoculation with *Meloidogyne graminicola*. (**a**) Venn diagram depicting common and unique DEGs (|log_2_ fold change| ≥ 1, FDR < 0.05) in response to *M. graminicola* infection in cultivars 9311, 1008, and 685. (**b**) Bar plot showing the number of DEGs identified in cultivars 9311, 1008, and 685.

Differentially expressed genes (DEGs) were identified in the resistant cultivar 685, the moderately susceptible cultivar 1008, and the highly susceptible cultivar 9311 after *M. graminicola* infection, compared to mock-inoculated samples, using an absolute value of |log_2_(fold change)| ≥ 1 and a false discovery rate (FDR) < 0.05 as thresholds. Venn diagram analysis showed 528 DEGs unique to cultivar 685, and 986 DEGs unique to cultivar 9311. Furthermore, 60 DEGs were shared between cultivars 9311 and 685 (Figure 3a). Gene expression analysis revealed 1363 DEGs in the highly susceptible cultivar 9311, with 364 genes upregulated and 999 genes downregulated. In the moderately susceptible cultivar 1008, 535 DEGs were identified after infection, with 501 (93.64%) DEGs upregulated and 34 (6.36%) downregulated. Additionally, 606 DEGs were detected in the resistant cultivar 685, with 480 (79.21%) DEGs downregulated and 126 (20.79%) upregulated (Figure 3b). 

**Figure 4:**
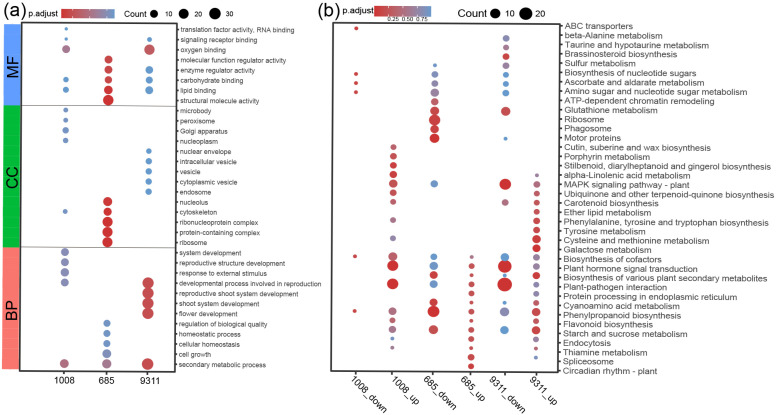
Gene enrichment analysis through the classification of gene ontology (GO) terms. (**a**) Gene ontology (GO) enrichment analysis of differentially expressed genes (DEGs) in rice cultivars 9311, 1008, and 685 during the early stages of *M. graminicola* infection. The top five significantly enriched GO terms are presented for molecular function (MF), cellular component (CC), and biological process (BP). (**b**) KEGG pathway analysis of DEGs in rice cultivars 685, 1008, and 9311 after *M. graminicola* inoculation. The top 10 significantly enriched KEGG pathways are shown.

Gene ontology (GO) analysis was conducted to identify the biological processes associated with the DEGs in the resistant cultivar 685, the moderately susceptible cultivar 1008, and the highly susceptible cultivar 9311 postinfection. DEGs were categorized into three primary GO terms: biological process (BP), cellular component (CC), and molecular function (MF) (Table S1). In cultivar 685, the predominant DEG annotations pertained to MF and CC classifications. Conversely, the DEGs in cultivar 9311 were primarily associated with BP terms (Figure 4a). Interestingly, the DEGs in cultivar 1008 did not show significant enrichment in any specific GO category (Figure 4a, Table S1). To further explore the biochemical pathways related to *M. graminicola* infection, Kyoto Encyclopedia of Genes and Genomes (KEGG) pathway enrichment analysis was performed using the KEGG automatic annotation server (KAAS) and assigned KO terms. The most significantly enriched KEGG pathways in response to *M. graminicola* infection were identified (Table S1). Among these, the “starch and sucrose metabolism” and “phenylpropanoid biosynthesis” pathways were enriched in downregulated DEGs in the resistant cultivar 685 and upregulated DEGs in the highly susceptible cultivar 9311 (Figure 4b). In addition,, the “plant–pathogen interaction” and “plant hormone signal transduction” pathways were significantly enriched in downregulated DEGs in the highly susceptible cultivar 9311 and upregulated DEGs in the moderately susceptible cultivar 1008. Notably, the “ribosome”, “motor proteins”, and “phagosome” pathways were uniquely enriched in downregulated DEGs in the resistant cultivar 685. Moreover, the mitogen-activated protein kinase (MAPK) signaling pathway was significantly enriched in downregulated DEGs in the highly susceptible cultivar 9311 (Figure 4b).

### 2.4. Weighted Gene Co-Correlation Network Analysis

**Figure 5:**
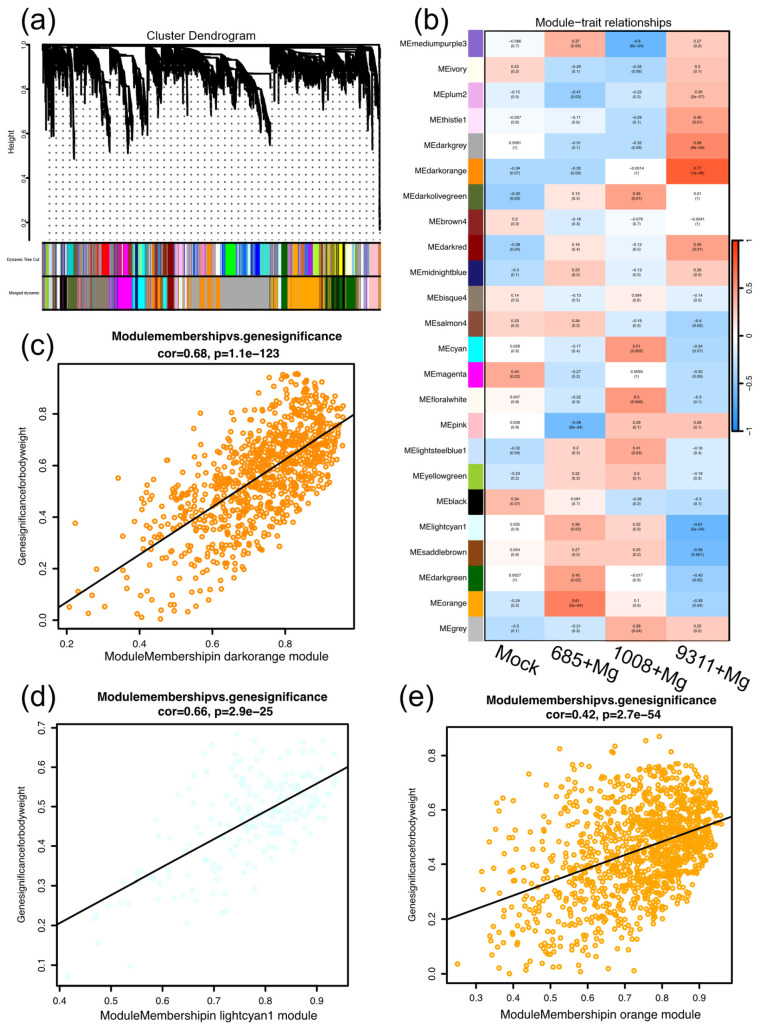
Co-expression network of expressed genes in rice cultivars following *M. graminicola* inoculation. (**a**) Hierarchical clustering tree of co-expressed genes identified by WGCNA. Each leaf represents a single gene, and the major branches form 24 distinct modules, each labeled with a different color. (**b**) Module–trait correlations in cultivars 685, 1008, and 9311 after *M. graminicola* infection. The numerical values represent the correlation coefficients between modules and phenotypic traits. The red module exhibits a positive correlation, whereas the blue module is negatively correlated. (**c**–**e**) Pearson’s correlation analysis between gene significance (GS) in cultivars 685 and 9311 and module membership (MM) in the darkorange, orange, and lightcyan1 modules.

To further investigate the regulatory network responses of resistant and susceptible rice cultivars to nematode infection and identify key genes associated with nematode resistance and susceptibility, WGCNA was performed. Genes with TPM ≥ 5 in at least one sample were included in the construction of a scale-free co-expression network, applying a soft-thresholding power of β = 10. WGCNA identified 24 modules, each represented by a distinct color, using the dynamic tree-cutting method (core parameter: MEDissThres = 0.25) (Figure 5a). Notably, 3 out of the 24 modules exhibited a strong correlation with traits related to disease resistance or susceptibility, using Pearson correlation coefficients (r > 0.6) as screening criteria. Among these, the darkorange module (728 genes; R^2^ = 0.77, *p* = 1.0 × 10^−6^) was strongly positively correlated with nematode infection in the susceptible cultivar 9311 (Figure 5b). Conversely, the lightcyan1 module (191 genes; R^2^ = −0.61, *p* = 5 × 10^−4^) showed a significant negative correlation with nematode infection in the same susceptible cultivar. In the resistant cultivar 685, the orange module (810 genes; R^2^ = 0.52, *p* = 2.7 × 10^−54^) was strongly positively correlated with nematode resistance (Figure 5b). The module membership (MM) and gene significance (GS) for nematode infection in the darkorange, lightcyan1, and orange modules are shown (Figure 5c–e).

### 2.5. Functional Analysis of Gene Modules Associated with Susceptibility and Resistance to M. graminicola

**Figure 6:**
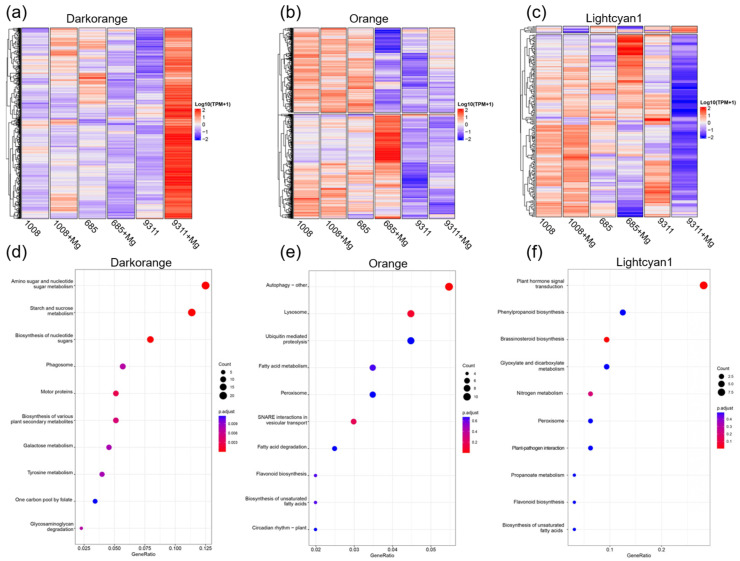
Functional analysis of genes in WGCNA modules. (**a**–**c**) Heatmap clustering of genes in the darkorange, orange, and lightcyan1 modules, illustrating gene expression differences between *M. graminicola*-infected and uninfected plants in cultivars 685, 1008, and 9311. (**d**–**f**) KEGG enrichment analysis of genes in the darkorange, orange, and lightcyan1 modules. The top 10 significantly enriched KEGG pathways are presented.

The darkorange, lightcyan1, and orange modules were identified as key regulators associated with resistance and susceptibility in cultivars 685 and 9311 and selected for in-depth analysis. Gene expression profiles within these modules were visualized using heatmaps to reveal their regulatory dynamics (Figure 6a–c). In the susceptible cultivar 9311, consistent upregulation of genes in the darkorange module was observed post-infection, whereas genes within the lightcyan1 module were predominantly downregulated under identical conditions (Figure 6a,c). In contrast, the resistant cultivar 685 displayed a divergent response, with more than half of the genes in the orange module exhibiting upregulation in response to nematode infection, while the remaining genes were downregulated (Figure 6b). To investigate the functional roles of genes within each module, KEGG pathway enrichment analysis was conducted. The darkorange module was primarily enriched in pathways related to sugar metabolism, such as “Amino sugar and nucleotide sugar metabolism”, “Starch and sucrose metabolism”, and “Biosynthesis of nucleotide sugars” (Figure 6d). This module included genes encoding key enzymes in sugar metabolism, including sucrose synthase genes (*OsSUS1*, *OsSUS4*), UDP-glucose pyrophosphorylase (*OsUgp1*), UDP-glucose 6-dehydrogenase, sucrose-phosphatase, sucrose-phosphate synthase (*OsSPS8*), and cytosolic phosphoglucomutase (*OscPGM*) (Table S1). The orange module was enriched in pathways associated with autophagy and intracellular trafficking, including “Autophagy-other”, “Lysosome”, and “SNARE interactions in vesicular transport” (Figure 6e). Several autophagy-related genes (*ATG101*, *ATG4b*, *ATG8a*, *ATG8b*, *ATG8c*, *ATG18a*, *Tap46*) were significantly upregulated in the cultivar 685 upon nematode infection, suggesting their involvement in enhancing resistance to *M. graminicola* (Table S1). In contrast, genes related to soluble N-ethylmaleimide-sensitive factor attachment protein receptor (SNARE) and lysosome pathways were significantly downregulated in cultivar 685 under the same conditions (Table S1). In the lightcyan1 module, enriched pathways were mainly linked to “Plant hormone signal transduction” and “Brassinosteroid biosynthesis” (Figure 6f). A set of genes functioning in the salicylic acid (SA) signaling pathway, particularly TGA transcription factors (*Os02g0194900*, *Os09g0280500*, *Os12g0152900*, *Os11g0152700*, *Os09g0489500*), were markedly downregulated in cultivar 9311 in response to nematode infection. Additionally, the key gene in BR biosynthesis, *Dtl2* (*Os01g0851600*), along with the downstream genes of cytochrome P450 monooxygenase (*CYP734A2_Os02g0204700* and *CYP734A5_Os07g0647200*), showed significant downregulation in cultivar 9311 upon infection. These results suggest a potential impairment in brassinosteroid-mediated defense signaling pathways in the susceptible cultivar.

### 2.6. Identification of Hub Genes Correlated with Susceptibility and Resistance to M. graminicola

**Figure 7:**
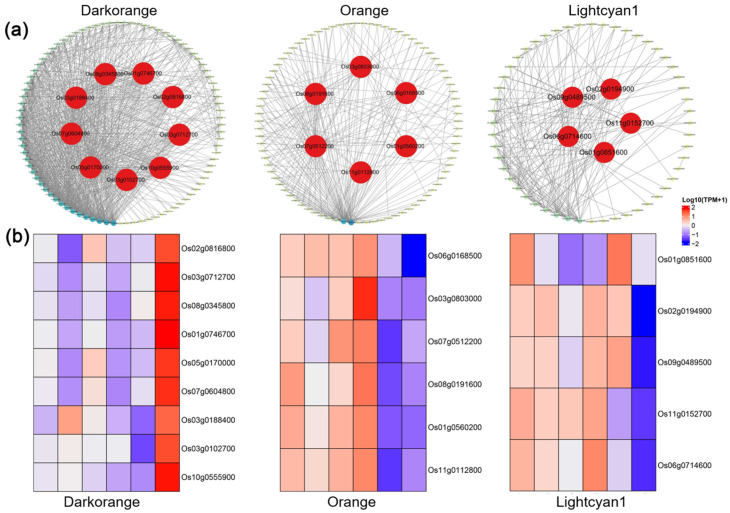
Co-expression network analysis of hub genes in the darkorange, orange, and lightcyan1 modules. (**a**) Co-expression network of hub genes with high connectivity in the darkorange, orange, and lightcyan1 modules. (**b**) Expression levels of hub genes in the darkorange, orange, and lightcyan1 modules (*p* < 0.05).

Strong correlations between the darkorange, orange, and lightcyan1 modules and transcriptional responses to nematode infection in rice cultivars 9311 and 685 were observed and thus analyzed in further analysis. The darkorange module contained 98 highly correlated genes, identified as potential regulators of susceptibility in cultivar 9311 (Figure 7a). Notably, *OsbHLH96* (*Os03g0188400*), a basic helix–loop–helix transcription factor critical for starch biosynthesis, emerged as a central hub gene strongly associated with nematode susceptibility. Other hub genes in the darkorange module, involved in sugar metabolism, included mitochondrial complex I subunit (*OsNDUFA9_Os02g0816800*), the small subunit of ADP-glucose pyrophosphorylase shrunken-2 (*OsAPS2_Os08g0345800*), UDP-arabinopyranose mutase 3 (*OsUAM3_Os07g0604800*), and cytosolic phosphoglucomutase (*OscPGM_Os03g0712700*) (Figure 7b). Genes related to cell wall sugar metabolism, such as OsMan02-Endo-Beta-Mannanase (*Os01g0746700*), β-expansin (*OsEXPB3_Os10g0555900*), and the type II membrane gene brittleculm 10 (*BC10*), exhibited high connectivity and were also identified as hub genes in this module (Figure 7b). The orange module, comprising 93 highly correlated genes, was linked to the nematode resistance response in cultivar 685 (Figure 7a). The key hub genes in this module, involved in the autophagy degradation pathway, included SNARE protein OsSYP131b (*Os06g0168500*), vesicle-associated membrane protein, and autophagy-related genes *OsATG8a* (*Os07g0512200*), *OsATG8c* (*Os08g0191600*), and saposin-like type B (*Os11g0112800*) (Figure 7b). In the lightcyan1 module, 59 highly correlated genes were identified as potential regulators of susceptibility in cultivar 9311 (Figure 7a). Among these, three basic leucine zipper (bZIP) transcription factors, *OsbZIP17* (*Os02g0194900*), *OsbZIP74* (*Os09g0489500*), and *OsbZIP79* (*Os11g0152700*), were recognized as central hub genes and key regulators of the SA signaling pathway (Figure 7b). Additionally, genes involved in BR biosynthesis, such as 3-oxo-5-alpha-steroid 4-dehydrogenase (*Os01g0851600*), and small GTP-binding protein OsPRA (Os06g0714600), were identified as hub genes (Figure 7b). Networks for each module were constructed using hub genes, visually represented as red nodes within the network.

### 2.7. Validation of Hub Genes by RT-qPCR

**Figure 8:**
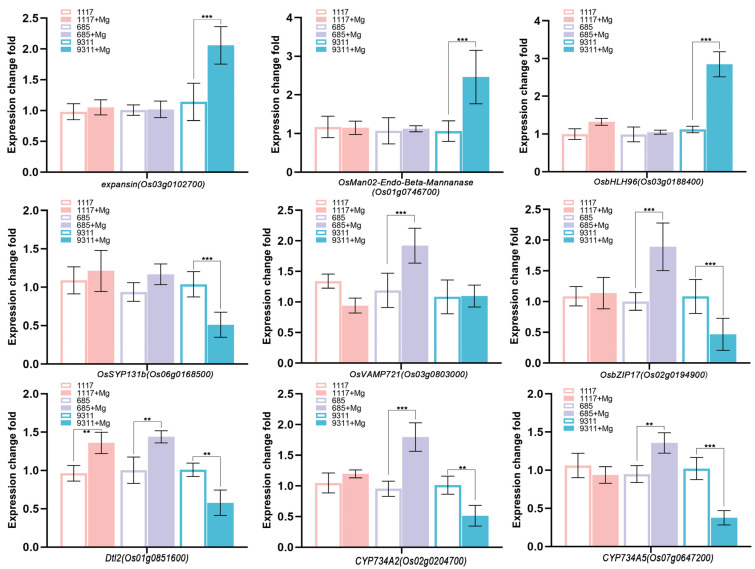
Validation of hub genes by quantitative real-time PCR (qRT-PCR). Expression validation of hub genes in the blue2 and pale turquoise modules in root tissues (*p* < 0.05). The relative expression levels were normalized using UBQ as the reference gene. The genes analyzed include *Os04g0413200*, *Os04g0413500*, *Os04g0535600*, *Os04g0664800*, *Os04g0664900*, *Os09g0255000*, and *Os09g0255266*. Data are presented as means ± SD from three plants across three independent biological replicates. ** *p* < 0.01, *** *p* < 0.001, Student’s *t*-test.

To validate the reproducibility and reliability of the RNA-seq dataset, representative genes from selected pathways and co-expression modules were subjected to qRT-PCR analysis (Figure 8). The average fold change in nine genes was illustrated in Figure 8. These findings suggest that the expression patterns of hub genes were consistent with RNA-seq data.

## 3. Discussion

### 3.1. Mining of M. graminicola Resistance Resources and Molecular Mechanism

Rice and other crops face persistent threats from diverse pathogens in their cultivation environments, among which plant-parasitic nematodes are recognized as some of the most damaging [26]. Over millions of years, plants, including rice, have co-evolved with these threats, developing a highly intricate innate immune system capable of pathogen recognition and resistance [12]. Although nematicides have been widely deployed for nematode management, their efficacy remains limited, and numerous compounds have been banned due to environmental toxicity. In contrast, disease resistance breeding, which exploits genetic resistance resources, has emerged as the most effective and ecologically sustainable strategy for long-term nematode control [27]. Natural resistance to *M. graminicola*, a highly virulent rice root-knot nematode, has been identified in Asian rice, African rice, and wild rice species [28,29]. In this study, 122 indica rice varieties were screened to identify resistant germplasms against *M. graminicola*. A notable finding was the identification of indica rice variety 685, which exhibits high resistance to *M. graminicola*. Further analysis of nematode development within the resistant 685 variety revealed the failure of *M. graminicola* to mature into females within the root tissue (Figure 2b). This suggests that variety 685 effectively disrupts the formation of nematode-induced feeding site cells at an early stage of infestation, thereby suppressing nematode development.

Although several rice germplasm accessions display resistance to *M. graminicola*, only one single resistance gene, *Mg1*, has been cloned to date [13]. The molecular basis governing resistance and susceptibility in rice, however, remains poorly understood. To elucidate the resistance mechanism against *M. graminicola*, RNA sequencing was employed to compare the gene expression profiles of highly resistant, moderately susceptible, and highly susceptible rice varieties at 5 dpi following nematode infection. GO analysis was conducted to categorize the biological processes associated with DEGs. In the resistant variety 685, DEGs were predominantly enriched in the CC and MF categories, whereas in the susceptible variety 9311, enrichment was primarily observed in the BP category. The enrichment of CC-related DEGs in the resistant variety suggests structural modifications at the cellular level, potentially contributing to physical or biochemical defenses against nematodes. The prevalence of MF-associated DEGs may indicate functional alterations in proteins involved in pathogen recognition and immune signaling. In contrast, the enrichment of BP-related DEGs in the susceptible variety likely reflects broader disruptions in biological processes, including signal transduction, metabolic reprogramming, and cell cycle regulation.

### 3.2. Sugar Metabolism Has an Important Role in Nematode–Host Interactions

KEGG enrichment analysis indicated the substantial downregulation of DEGs involved in the starch and sucrose metabolism pathway in nematode-infected resistant rice variety 685, in contrast to a marked upregulation observed in the susceptible rice variety 9311 (Figure 4b). This trend was further corroborated by WGCNA, in which the darkorange module in the KEGG analysis demonstrated a positive correlation between nematode infection and sugar metabolism in susceptible rice variety 9311. Enriched pathways included “Starch and sucrose metabolism”, “Amino sugar and nucleotide sugar metabolism”, and “Biosynthesis of nucleotide sugars” (Figure 6d). Starch and sucrose, as primary carbohydrates in plants, are key regulators of plant development, responses to abiotic stress, and plant–microbe interactions. Previous studies have reported a substantial increase in sucrose and starch levels within syncytia compared to uninfected roots during *Heterodera schachtii* infection in *Arabidopsis* [30,31]. Extensive studies have demonstrated a significant modification of starch and sucrose metabolism at 7 and 14 dpi. In root-knot nematode-induced galls, starch accumulation increased threefold, suggesting its role as a carbohydrate reservoir to support nematode development [32]. Enzymatic assays further confirmed pronounced differences in starch content between galls and uninfected roots, reinforcing the metabolic adaptations in nematode-feeding sites [32]. These alterations in primary metabolism within galls resemble those observed in syncytia, indicating shared functional mechanisms despite distinct developmental origins [33].

Sucrose metabolism is central to energy production, macromolecule biosynthesis, and amino acid biosynthesis and is predominantly regulated by the invertase (INV) and sucrose synthase (SUS) enzyme families [34]. Transcriptomic profiling analysis revealed significant differential expression of sucrose synthase (Sus) (EC 2.4.1.13) genes between resistant and susceptible rice varieties. The key genes *SUS3* (*Os07g0616800*), *SUS4* (*Os03g0340500*), *SUS5* (*Os04g0309600*), *SUS6* (*Os02g0831500*), and *SUS7* (*Os04g0249500*) were specifically upregulated in the susceptible rice cultivar 9311, while their expression remained largely unchanged in the moderately susceptible cultivar 1008 (Table S1). In contrast, a significant downregulation of *Sus* genes was detected in the resistant cultivar 685. Furthermore, among the 19 rice INV genes, eight—*Os04g0664900*, *Os01g0966700*, *Os11g0173600*, *Os04g0405500*, *Os04g0413500*, *Os02g0534400*, *Os09g0255000*, and *Os02g0529400*—displayed strong upregulation in cultivar 9311 (Table S1). The expression patterns of *Sus* and *INV* genes were closely associated with rice susceptibility to nematode infection.

Recent investigations have highlighted the pivotal role of sugar transporters in plant–pathogen interactions [35]. *OsSWEET14*, encoding a sucrose transporter, has been shown to influence disease resistance in a context-dependent manner: its knockout enhances resistance to bacterial leaf blight, whereas its overexpression confers improved resistance to sheath blight [36,37]. Similarly, *OsSWEET12* and *OsSWEET13* function as susceptibility genes for bacterial leaf blight, facilitating pathogen-induced symptom development [38,39]. In plant–nematode interactions, *AtSWEET1* modulates host susceptibility to root-knot nematodes [40]. Other sugar transporter genes, such as *OsSUT1* and *OsSUT2*, are induced at brown planthopper (BPH) oviposition sites and are associated with callose deposition, suggesting a distinct role in *M. graminicola* infection [41]. Moreover, *AtSUC2* and *AtSUC4* are expressed within syncytial feeding structures, and RNAi-mediated silencing of *AtSUC4* significantly reduces female nematode numbers, underscoring the importance of sucrose transporters in nematode development [30]. In this study, gene expression analysis revealed that the sucrose transporter genes *OsSWEET1a* (*Os01g0881300*) and *OsSWEET13* (*Os12g0476200*) were actively upregulated upon nematode infection in the susceptible rice variety 9311, whereas no significant changes were observed in the resistant variety 685 (Table S1). As primary carbohydrates, starch and sucrose serve as essential energy sources and structural components, fulfilling the nutritional demands of nematodes. The suppressed expression of sugar metabolism-related genes in the resistant line 685 may contribute to the restriction of nematode development. Nonetheless, the precise role of sucrose and starch at nematode feeding sites and plant–nematode interactions, particularly in immune responses, remains largely unexplored.

### 3.3. Autophagic Degradation Pathway Regulates Plant-Nematode Immune Interactions

Autophagy constitutes a key regulatory mechanism in maintaining plant homeostasis, development, senescence, and responses to both abiotic and biotic stresses. Notably, it is increasingly recognized as an integral component of plant immunity, functioning effectively against viral, bacterial, and filamentous pathogens. Despite this, research on the interplay between plant-parasitic nematodes and host cell autophagy remains poorly understood. In *Arabidopsis thaliana*, loss-of-function mutations in the autophagy-related genes *atg8f* and *atg8h* resulted in a significant increase in nematode susceptibility, suggesting that nematode parasitism depends on a finely regulated balance of programmed cell death (PCD) factors [42]. During host–nematode molecular interactions, the potato golden nematode has been shown to exploit the effector protein NMAS1 to specifically target the host autophagy core protein ATG8 via its AIM domain, effectively suppressing host immunity and facilitating nematode parasitism [43]. Additionally, Zou et al. demonstrated that plant cell autophagy enhances jasmonic acid (JA)-mediated defense against the southern root-knot nematode by regulating the degradation of JA pathway proteins and JAMs and promoting ERF1 transcriptional activation [44]. These results highlight the critical role of autophagy in modulating plant–nematode interactions. In this study, gene expression analysis of the orange module in WGCNA revealed a significant upregulation of autophagy-related genes, including *ATG8a*, *ATG8c*, *ATG101*, and *Tap46*, in the resistant cultivar 685 (Table S1). This suggests that autophagy, particularly its role in nematode resistance, is a key defense mechanism in 685. Interestingly, the SNARE gene *OsSYP131b*, which regulates amphisome formation by mediating the fusion of autophagosomes with endocytic structures, was significantly downregulated in resistant cultivar 685. SNARE-mediated immune secretion is a key component of plant immunity, with AtSYP121 being essential for resistance against fungal and oomycete pathogens and NbSYP132 being involved in bacterial defense responses [45]. In rice, the simultaneous loss of OsSYP131b and OsSYP132 strongly suppresses arbuscular mycorrhizal (AMF) colonization in roots [46]. However, the specific role of SNAREs in plant–nematode immune interactions remains unclear and warrants further investigation.

### 3.4. M. graminicola Regulate the Salicylic Acid (SA) and Brassinosteroid (BR) Signaling Pathway to Promote Nematode Parasitism

Phytohormone homeostasis has been increasingly recognized as a key determinant in shaping plant–pathogen interactions. In the early stages of nematode infection, significant downregulation of multiple genes associated with the phenylpropanoid pathway was observed. This essential metabolic pathway coordinates the biosynthesis of key plant defense compounds, including lignin precursors, flavonoids, hydroxycinnamic acid esters, and salicylic acid (SA) [47]. SA plays a pivotal role in plant immunity, particularly in pathogen resistance and systemic acquired resistance, primarily mediated through a nonexpressor of pathogenesis-related genes (NPRs) and TGACG motif-binding transcription factors (TGAs). Transcriptome analysis of all 12 TGA gene family members in rice revealed significant downregulation in the susceptible cultivar 9311 following nematode infection, suggesting a regulatory function for TGAs in plant–nematode interactions (Table S1). Functional studies indicate that TGAs are sensitive to cellular redox states and participate in pathways mitigating reactive oxygen species (ROS) accumulation under stress conditions [48]. Additionally, TGA transcription factors play a critical role in defense against biotic stress, particularly in resistance-gene-mediated immunity. For instance, TGA transcription factors are involved in Pto-mediated resistance to *Pseudomonas syringae* pv. tomato, and TGA1a in tomato has been shown to mediate *Mi-1*-dependent resistance to *Meloidogyne javanica* [49,50]. Although TGA TFs are integral to plant defense, the specific mechanisms through which nematodes suppress the rice immune system remain to be elucidated.

Beyond immune regulation, TGAs also participate in multiple phytohormonal signaling pathways, such as BR signaling. Previous RNA-seq data from rice indicated that the BR pathway is generally significantly induced at 7 days after inoculation (dai) in RKN gall tissue, but this study shows an early downregulation of the BR pathway upon RKN infection [47]. BRs have been shown to enhance SA-mediated immune responses by inhibiting the phosphorylation of clade I TGA transcription factors via BIN2 in Arabidopsis, implicating TGAs in BR signaling [51]. In this study, all three CYP734A genes (*Os02g0204700*, *Os07g0647200*, *Os06g0600400*) were significantly suppressed in the susceptible cultivar 9311 upon nematode infection. In rice, CYP734As function as key regulators of endogenous BR levels, inactivating bioactive BRs while suppressing their biosynthesis by modulating brassinosteroid precursor concentrations [52]. These results suggest that nematodes may enhance BR accumulation by downregulating CYP734A gene expression. Notably, endogenous BRs antagonize the JA-mediated defense response, thereby suppressing rice roots’ innate immunity against *M. graminicola* [53]. These results indicate that nematodes may exploit CYP734A suppression to inhibit BR degradation, thereby attenuating JA-mediated defense responses and promoting parasitism.

This study identifies key resistance resources in rice against *M. graminicola* and delineates differential transcriptional regulatory networks between the resistant cultivar 685 and the susceptible cultivar 9311 under a nematode challenge. A comprehensive analysis of gene expression profiles highlighted critical pathways, including sugar metabolism, autophagy, and SA- and BR-mediated signaling. The key genes implicated in nematode susceptibility and resistance, including Suc genes, sugar transporter genes, autophagy-associated genes, TGA genes, and CYP734A genes, were identified and validated. Further studies employing genetic and molecular approaches are necessary to determine the functional contributions of these candidate genes to rice resistance and susceptibility to *M. graminicola*.

## 4. Materials and Methods

### 4.1. Plant Material and Nematode Inoculation

The rice seeds were germinated in an incubator at 30 °C for 3–5 days before being transferred to a sand substrate for continued growth in a controlled growth chamber maintained at 26–28 °C under a 16 h light/8 h dark photoperiod. The *M. graminicola* population was collected from rice-growing regions in Guangzhou, China. Nematodes were purified from individual egg masses and maintained in a sterilized sand–soil mixture (3:1) at 26–28 °C, with *Oryza sativa* cv. Nipponbare serving as the host. Pre-parasitic second-stage juveniles (pre-J2s) were collected following the protocol described by Chen et al. [10].

### 4.2. Nematode Infection Experiments

To evaluate rice varietal resistance to *M. graminicola*, each two-week-old rice seedling was inoculated with 200 s stage juveniles (J2s). At 20 days post-inoculation, root systems were carefully extracted. For the female nematode assay, gall formation was visualized via acid fuchsin staining. Rice roots were boiled in 0.8% (*v*/*v*) acetic acid and 0.013% (*w*/*v*) acid fuchsin for 5 min, followed by destaining in acidified glycerol for 3–4 days. Stained roots were examined under a stereomicroscope. Experiments were conducted in triplicate, with five biological replicates per treatment.

For RNA-seq analysis, two-week-old rice seedlings were inoculated with 200 J2s, while control plants were mock-inoculated with water. At three days post-infection, root tissues—including nematode-infected and control roots—were collected. Nematode-infected root segments were harvested 0.5 cm away from the infection sites, and equivalent regions were sampled from mock-inoculated controls. Root materials from 30 plants were pooled per sample, with five independent biological replicates analyzed.

### 4.3. RNA-Seq and Differentially Expressed Gene Analysis

Total RNA was extracted from these biological replicates using TRIzol reagent (Invitrogen, Waltham, MA, USA), following the manufacturer’s protocol. mRNA was purified from total RNA using magnetic beads (NEB Next Poly(A) mRNA Magnetic Isolation Module) and used for sequencing library construction with the NEBNext Ultra RNA Library Prep Kit (NEB, Ipswich, MA, USA). First-strand cDNA synthesis was performed using random hexamer primers and M-MuLV reverse transcriptase (NEBNext Ultra™ II RNA Library Prep Kit), followed by second-strand synthesis using DNA polymerase I (NEBNext Ultra™ II RNA Library Prep Kit). cDNA fragments of 250–300 bp were selected and purified using AMPure XP beads with a double-sided bead cleanup protocol (Beckman Coulter, Carlsbad, CA, USA). Sequencing was performed on the Illumina HiSeq platform, generating 125 bp/150 bp paired-end reads. Low-quality reads, adapter sequences, and poly-N reads were removed using Cutadapt (v4.7) to obtain high-quality clean reads. GC content was assessed, and all downstream analyses were based on these filtered datasets. Clean reads were aligned to the rice reference genome (IRGSP-1.0) using HISAT2 v2.2.1. Transcript assembly was conducted with StringTie v2.1.7, and transcript abundance was quantified as transcripts per million (TPM) based on gene length and mapped read counts.

### 4.4. Weighted Gene Co-Expression Network Analysis

To elucidate the complex interplay between genes and phenotypic traits, WGCNA was applied to genes exhibiting transcripts per million (TPM) values exceeding 5 in at least one sample. The WGCNA package (v1.72.5) in R was employed to construct gene networks and identify modules associated with resistance or susceptibility to *M. graminicola* in rice cultivars. An unsigned topological overlap matrix (TOM) was used for network construction and module detection, with key parameters set as follows: soft-thresholding power (β) = 10 (scale-free topology fit > 0.85). Module identification was performed using hierarchical clustering with the hclust function (method = “average”), followed by dynamic tree cutting using cutreeDynamic with the following parameters: minimum module size = 30, depth split = 2, and minimum height threshold for module merging = 0.25. Module eigengenes (MEs) were computed to assess correlations between gene modules and lesion diameter. Module–trait relationships were evaluated by correlating MEs with phenotypic traits, and key genes within each module were identified based on gene significance (GS) and module membership (MM) values. Interaction networks comprising the top ten hub genes were constructed and visualized using Cytoscape (v3.9.1) using edge weights derived from TOM similarity (edgeThreshold = 0.02).

### 4.5. Differential Gene Expression Analysis and Gene Enrichment Analysis

Differential gene expression analysis was performed using the DESeq2 R package (v1.20.0), applying a log_2_ fold change ≥ 1 and an FDR < 0.05 as significance thresholds. Raw counts were modeled using a negative binomial distribution with the DESeq2 function, which incorporated normalization via the median-of-ratios method (estimateSizeFactors) and dispersion estimation (estimateDispersions). Differential expression was tested using the Wald test (nbinomWaldTest), with results extracted using the results function at thresholds of |log_2_ fold change| ≥ 1 and false discovery rate (FDR) < 0.05 (Benjamini–Hochberg correction). Functional enrichment analysis was conducted using clusterProfiler (v4.0). Gene Ontology (GO) terms were analyzed using enrichGO with Benjamini–Hochberg correction (pAdjustMethod = “BH”) and significance thresholds of *p* < 0.05 and *q* < 0.1. KEGG pathway enrichment was performed using enrichKEGG with organism = “dosa”.

### 4.6. Validation of Genes by qRT-PCR

Total RNA was extracted from the roots of two-week-old rice seedlings, both inoculated with nematodes and mock-inoculated controls, using the RNAprep Pure Micro Kit (Tiangen Biotech, Beijing, China). First-strand cDNA synthesis was carried out with the TransScript All-in-One First-Strand cDNA Synthesis SuperMix (TransGen Biotech, Beijing, China) following the manufacturer’s protocol. qRT-PCR was performed using gene-specific primers (Table S1), with *OsUBI* serving as the endogenous reference gene for normalization [10]. The primer sequences used for qRT-PCR are detailed in Table S1.

## 5. Conclusions

This study provides a comprehensive transcriptomic analysis of three rice cultivars exhibiting distinct resistance or susceptibility to *M. graminicola*, revealing differential responses to nematode infection. WGCNA identified key pathways and modules predominantly involved in sugar metabolism, autophagy, and phytohormone signaling (SA and BR). The identification of hub genes and regulatory pathways offers critical insights into the molecular basis of nematode resistance and susceptibility, providing a foundation for targeted genetic improvement in rice breeding programs.

## Data Availability

The datasets are available in the NCBI Sequence Read Archive (PRJNA1240874).

## Supplementary Materials

The following supporting information can be downloaded at https://www.mdpi.com/article/10.3390/ijms26115315/s1, Table S1.

## Abbreviations

The following abbreviations are used in this manuscript:

DEGs	Differential expressed genes
GCs	Giant cells
RNK	Root-knot nematode
WGCNA	Weighted gene co-expression network analysis
SCN	Soybean cyst nematode
GO	Gene ontology
KEGG	Kyoto Encyclopedia of Genes and Genomes
HR	Hypersensitive response
